# Dysregulated neuronal mRNA transport and translation in FTD/ALS

**DOI:** 10.1038/s44400-026-00116-5

**Published:** 2026-07-08

**Authors:** Emma J. Craig, Veronica H. Ryan

**Affiliations:** Center for Alzheimer’s and Related Dementias (CARD), National Institute on Aging, National Institutes of Health, Bethesda, MD USA

**Keywords:** Neurology, Neuroscience

## Abstract

Frontotemporal dementia (FTD) and amyotrophic lateral sclerosis (ALS) are fatal, co-occurring neurodegenerative disorders. Dysregulation of mRNA metabolism, transport, and local translation is a significant mechanism contributing to FTD/ALS. Here, we review the processes of neuronal RNA transport and translation, their disruption in FTD/ALS, and mechanistic interplay between the two. Finally, we discuss current progress targeting transport and translation defects and identify potential future directions for therapeutic development.

## Introduction

Frontotemporal dementia (FTD) and amyotrophic lateral sclerosis (ALS) are devastating neurodegenerative disorders characterized by progressive motor and cognitive dysfunction. FTD is the third most common form of dementia and manifests as profound changes in language, behavior, and executive function due to degeneration of the frontal and temporal lobes^1,2^. ALS causes loss of upper and lower motor neurons, leading to muscle weakness, paralysis, and ultimately respiratory failure, with a median survival of 3 to 5 years post-diagnosis^3^. Although they represent distinct clinical syndromes, FTD and ALS often co-occur, with at least 50% of ALS patients exhibiting cognitive symptoms associated with FTD, and around 25% of FTD patients showing motor dysfunction^4–7^. Because of this clinical overlap, as well as overlapping genetic causes and disease mechanisms, we will refer to these diseases as FTD/ALS here. Some drugs exist which slow disease progression, extend life expectancy, and manage symptoms, although they do not cure either condition^8–11^. Given the urgent need for an effective treatment, understanding the pathological mechanisms behind FTD/ALS and developing strategies to target them could offer a promising avenue for therapeutic development.

**Fig. 1:**
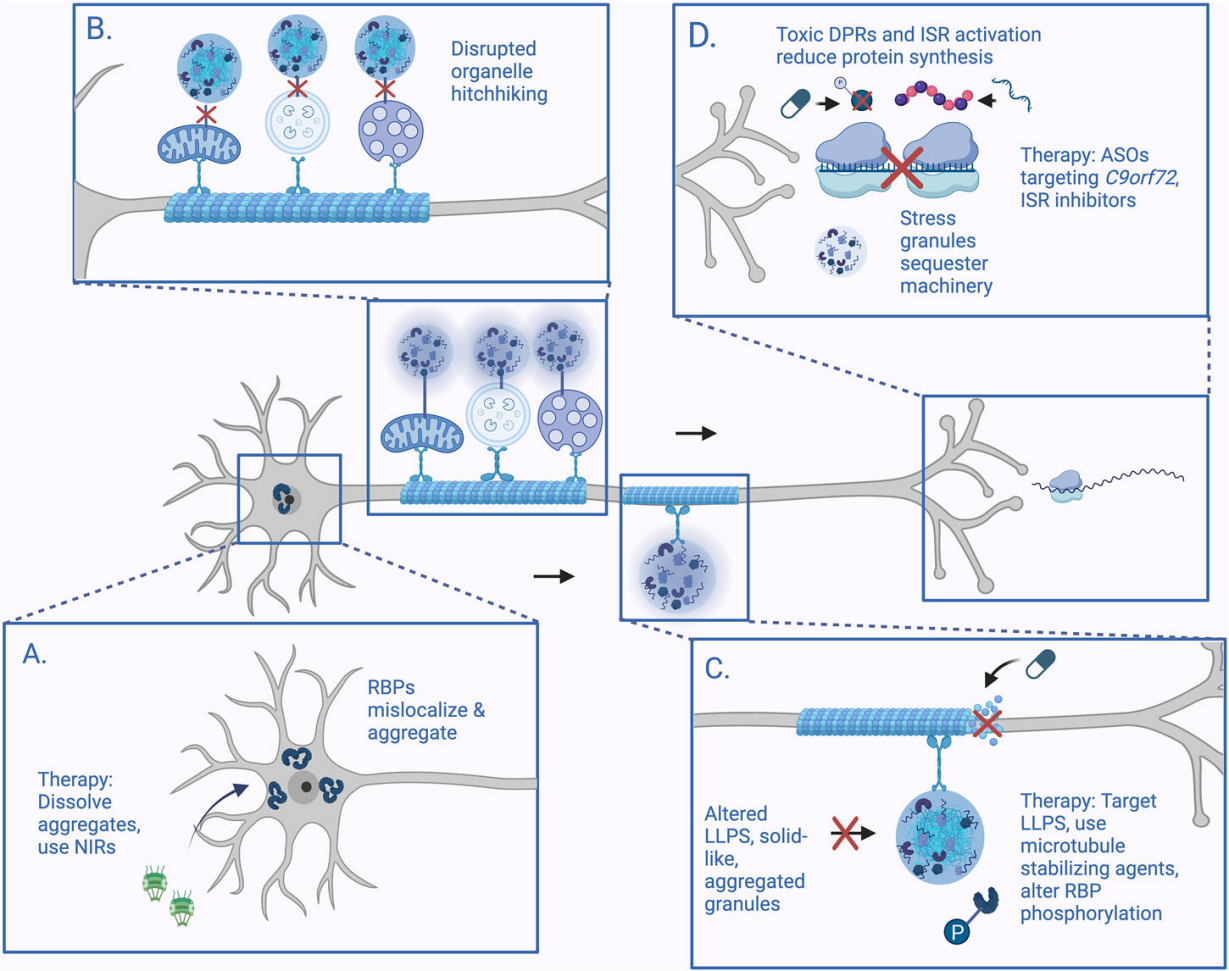
Summary of mRNA transport, local translation, and therapeutic targeting in FTD/ALS. **A** In the soma, normally nuclear RBPs become mislocalized and aggregated in the cytoplasm, which may be targeted by dissolving aggregates or employing nuclear import receptors (NIRs). **B** mRNA is transported via hitchhiking with organelles along the axon, which is disrupted in FTD/ALS. **C** RBPs, mRNA, and proteins are transported in liquid-like granules down the axon, transported by motor proteins. Altered phase separation yields solid-like, less mobile granules, which may be corrected by targeting LLPS directly, applying microtubule stabilizing agents, or altering phosphorylation of relevant RBPs. **D** Local translation in the neurites is reduced due to DPR expression, activation of the ISR, and because translation machinery is sequestered in stress granules. These defects may be treated using ASOs targeting *C9orf72* or using partial inhibitors of the ISR. Created in BioRender. Craig, E. (2026) https://BioRender.com/0901n0u

A potential mechanism underlying these diseases is the dysregulation of RNA metabolism, transport, and local translation. Neurons are large, polarized cells composed of soma (the cell body) and neurites (distal neuronal compartments, including the axons and dendrites). Given their size, it is energetically costly and temporally inefficient to transport proteins to the neurites; as such, they rely on mRNA transport and local translation to regulate localized protein production and maintain neuronal health^12–14^. In healthy cells, motor proteins, organelle hitchhiking, RNA binding proteins (RBPs), and transport granules ensure that these RNA transcripts are localized to and translated in the correct places^15,16^. In FTD/ALS, however, these mechanisms become disrupted, and impaired mRNA transport and local translation are associated with neuronal degeneration (Fig. 1). Key players regulating both mRNA transport and translation are RBPs. RBPs are involved in nearly every component of RNA metabolism, including transport, translation, and alternative splicing. Several FTD/ALS-associated genes encode RBPs, and mutations in and dysregulation of these proteins lead to the loss of transcript stability, mislocalization, protein aggregation, and impaired RNA processing, representing a common pathological feature behind multiple neurodegenerative diseases^17–20^. Moreover, RBP dysfunction and other factors lead to a loss of translational control in FTD/ALS^21^. These findings indicate a central role of RNA in the pathology of FTD/ALS, making it a compelling target for therapeutic intervention.

In this review, we explore how dysregulation of RNA transport and translation contribute to disease pathology. We examine the processes of transport and translation in healthy neurons, their disruption in FTD/ALS, and their contributions to disease pathology. Finally, we highlight current translational progress targeting transport and translation defects and identify potential targets for future development.

## RNA transport mechanisms and dysregulation in FTD/ALS

### Neuronal RNA transport

In healthy neurons, several mechanisms regulate the localization of mRNA transcripts. Short zipcode sequences in the primary structure of mRNA direct localization. The first of these characterized was the β-actin mRNA zipcode, which is a short 54-nucleotide sequence located in the 3′ untranslated region (UTR)^22^. This sequence forms secondary structures which enable binding of relevant proteins, and together with its protein interactor, zipcode binding protein 1 (ZBP1, also called IGF2BP1), this sequence directs β-actin mRNA to the growth cone and inhibits translation until reaching its destination^23,24^. Recent methodological advances have identified several hundred additional 3′ UTR zipcode sequences, although more work is needed to characterize the exact roles and interactors of these novel zipcodes^12,25^. In addition to zipcode sequences, neuronal transcripts localized to distal sites like axons and dendrites may have distinct characteristics compared to those localized to the soma. Several studies document longer, more stable neuritic transcripts and UTRs^26–29^, and specific protein binding sites on these 3′ UTRs help guide transcript localization. These localization motifs often enable proper localization of specific isoforms formed by alternative 3′ UTR splicing and polyadenylation^30^. Additionally, multiple localization motifs may work combinatorially, with any single motif being insufficient to induce localization to the neurites^31^. Altogether, regulatory elements within untranslated and coding regions initiate the process of mRNA localization, which ultimately informs the local proteome^32^.

**Fig. 2:**
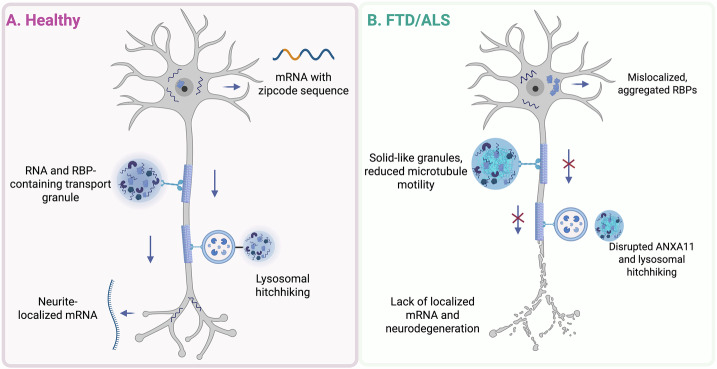
mRNA transport in healthy and FTD/ALS neurons. **A** RNA transport in healthy neurons. mRNA transcripts containing zipcode sequences and localization motifs are directed down the axon as part of liquid-like transport granules, where they may be transported using molecular motors or by hitchhiking on organelles to the neurites. **B** In FTD/ALS, RBPs become mislocalized to cytoplasmic aggregates. Transport granules lose liquid-like properties and motility. Disruptions to ANXA11 tethering alters lysosomal hitchhiking, leading to altered neuritic transcriptomics and axonal degeneration. Created in BioRender. Craig, E. (2026) https://BioRender.com/2ll1ht5

Due to their length and the slow diffusion rate of passive transport, neuronal mRNA is actively transported by molecular motors, including kinesin, dynein, and myosin^33–37^. KIF5A, for example, a kinesin which has RBP-like properties, directly binds to transcripts encoding synaptic proteins to guide their localization^38^. To facilitate transport, mRNA, RBPs, and other proteins are packaged into transport granules, which form distinct compartments from the surrounding cytoplasm, called membraneless organelles, through liquid-liquid phase separation (LLPS)^39,40^. This phase separation is involved in both localization and translational control, processes which are vulnerable to dysregulation in diseased states (Fig. 2; reviewed in ref. ^41^). Some transport granules move via co-trafficking or “hitchhiking” with organelles like lysosomes, endosomes, and mitochondria^42–44^. In mammalian neurons, RNA is co-trafficked bidirectionally along axons with lysosomes, linked by the membrane-granule tethering factor ANXA11^45,46^. Similar findings have been documented for mitochondria, with the PINK1 mitochondrial mRNA being trafficked with mitochondria due to an association with the mitochondrial protein SYNJ2A^47^. These mechanisms tightly control neuronal transport and are critical to maintaining neuronal health.

### RBPs in RNA transport and localization

RBPs play crucial roles in the transport and localization of mRNA. A growing body of evidence supports an essential role of RBPs in the local transcriptome, including the TAR DNA-binding protein 43 kDa (TDP-43), fused in sarcoma (FUS), and heterogeneous nuclear ribonucleoprotein A1 and A2 (hnRNPA1/hnRNPA2). For example, TDP-43 regulates the balance of anterograde and retrograde transport through interactions with kinesin and dynein, as well as binding directly to mRNAs and other RBPs in transport granules^48^. Furthermore, TDP-43 knockdown disrupts the transport of transcripts encoding ribosomal proteins^49^ and alters the subcellular transcriptome, including in the axons and somatodendritic compartment^50^. Knockdown of either TDP-43 or hnRNPA1 leads to distinct neuritic transcriptomes compared to nontargeting knockdown cells^14^, while depletion of FUS or expression of pathogenic variants of hnRNPA1 and hnRNPA2 causes impaired localization of mRNA to the dendrites^51^. Finally, the RBP-like qualities of KIF5A are disrupted in ALS, increasing the accumulation of synaptic proteins, impairing mRNA binding, and altering stress responses^38^. Although these advances provide insight into the role of RBPs in mRNA localization and transport, research into the binding patterns of specific RBPs in brain-relevant cell types is still limited. Novel methods, however, including STAMP, TRIBE, and eCLIP, have begun to make progress on this topic more accessible^52–55^.

Notably, dysfunction and mislocalization of these RBPs are associated with FTD/ALS pathology. The majority of FTD/ALS cases (approximately 70% of FTD and 80% of ALS) are sporadic, meaning they occur without a family history^56,57^. A small subset of sporadic and familial cases is caused by mutations, including in genes encoding RBPs, which are typically autosomal dominantly inherited^56,57^. However, most FTD/ALS cases are linked to RBP proteinopathy^58^. TDP-43 is implicated in multiple neurodegenerative diseases^59,60^ and is the most common proteinopathy in FTD/ALS, observed in up to 97% of ALS patients and 45% of FTD patients^61^. Although rare cases are caused by *TARDBP* mutations (roughly 1 to 5%), the majority of FTD/ALS cases have TDP-43 pathology without a TDP-43 mutation or with a mutation in another gene. TDP-43 proteinopathy causes mislocalization of TDP-43 from the nucleus, disrupted LLPS, and formation of aggregates in cortical and spinal motor neurons^58,62–66^. Interestingly, the aggregates of TDP-43 associated with different subtypes of FTD seem to have different folds^67^ and interaction partners (i.e., ANXA11-TDP-43 co-aggregates in FTD type C^68,69^ are different from those in type A^70^). Beyond sequestration of ANXA11, another protein important for mRNA transport, the distinct effect of these different aggregates on RNA biology is unknown, as is whether all types of TDP-43 pathology have co-aggregates, although some seem to be only TDP-43^71^. Mutations in FUS are unique among mutations in RBPs in that they do not cause TDP-43 proteinopathy^66^, but like TDP-43, FUS is normally nuclear and becomes mislocalized to the cytoplasm in conditions of stress or disease^72,73^. Interestingly, in FTD cases with FUS mutations, FUS itself does not aggregate, but a family member named TAF15 forms amyloid-like fibrils instead, pathologically distinguishing it from ALS^74,75^. hnRNPA1 and hnRNPA2 also each have rare genetic mutations associated with FTD/ALS, accounting for less than 1% of cases^72,73^. The effects of hnRNPA1 and hnRNPA2 mutations are not well characterized, although mutations may lead to misfolding, accumulation, and mislocalization^72,73,76,77^. Several other RBPs, including MATR3 and TIA1, are also implicated in FTD/ALS in association with TDP-43 proteinopathy^78,79^ and form aggregates under stress conditions. Another FTD-associated protein, tau, exhibits proteinopathy with similar effects, including aggregation, mislocalization, and disrupted axonal transport by altering microtubule stability^80,81^. Tau pathology accounts for most of the remaining FTD cases that do not have TDP-43 or FUS proteinopathy. Finally, hexanucleotide repeat expansion in *C9orf72*, the most common genetic cause of FTD/ALS, exhibits TDP-43 proteinopathy and pathology^82–84^. Notably, much of the mechanistic work on RNA metabolism in FTD and ALS has been done on cell culture or in vitro models that do not exhibit pathology similar to what is observed in patients (i.e., aggregates and different structures thereof). As such, these models cannot distinguish between FTD, ALS, or subtypes of either disease. Thus, these findings about phase separation, mislocalization, and protein-protein interactions may be relevant for both diseases. Regardless, the extensive association of RBPs with FTD/ALS suggests that disruption of the processes they regulate, including mRNA localization and transport, may contribute to FTD/ALS pathology, although more work is needed to establish a causal relationship.

FTD/ALS-associated mutations in RBPs may contribute to disease phenotypes via their disruption of transport granules. In healthy cells, transport granules have dynamic, liquid-like properties which enable correct transport in association with motor proteins and organelles. Disruption of these properties and resulting transport are implicated in several neurodegenerative diseases (reviewed in ref. ^85^). For example, mutant FUS disrupts transport by interfering with the normal function of kinesin and sequestering specific mRNA transcripts^86,87^. Furthermore, TDP-43 and FUS mutations are associated with disruption of the liquid-like properties and function of transport granules, altered granule phase separation, and formation of toxic, solid-like aggregates^88–92^. Trafficking of mRNA along axons is also disrupted in cells with mutant TDP-43 and FUS, resulting in impaired morphology and function in the neurites^93–96^. Intermediate polyglutamine expansions in Ataxin-2, another RBP, sequester TDP-43 in Ataxin-2 positive granules, disrupting their liquid-like properties and motility^97^. Similarly, hexanucleotide repeat expansion in *C9orf72* has been linked to impaired motility of transport granules along axons, likely due to a disruption in microtubule-based transport^98^. Furthermore, repeat expanded *C9orf72* is associated with disruptions to LLPS, likely interfering with the liquid-like quality of granules and impairing transport^99^. Lastly, mutations in the lysosome-RNA granule adapter protein ANXA11 lead to inclusions in patients and postmortem tissue, including co-fibrils with TDP-43^45,68,100^. Disease mutations alter phase changes in ANXA11-positive granules, suggesting a possible mechanism behind disrupted RNA trafficking and pathology in FTD/ALS patients with ANXA11 inclusions^101^. Despite this progress, the study of transport granule dynamics in FTD/ALS is a relatively new field, and more work is needed to determine the exact mechanisms behind this phenomenon and whether it contributes to disease phenotypes.

## Translation machinery and loss of translational control in FTD/ALS

### Local translation machinery in neurons

Once transported mRNA has reached its destination, specialized neuronal machinery enables local translation in the axons and dendrites (reviewed in ref. ^102^). Like in other cell types, ribosomes are the centerpiece of translation in neurons. Ribosome assembly occurs primarily in the nucleolus, and then they are transported to distal sites via association with RBPs in transport granules, endolysosomal hitchhiking, or cotransport with signaling machinery^103–107^. In neurites, ribosomes can be modified for specific tasks by locally translated ribosomal proteins that bind to distal ribosomes independently of the nucleolus^108^. Furthermore, ribosome heterogeneity, or specialization of translation machinery between ribosomes^109,110^ may contribute to ribosome specialization and neuron-specific local translation. A unique constraint of the neurites is the small size of these compartments. As such, polysomes, large structures formed from multiple ribosomes, are less abundant in neurites than in the soma. It was previously hypothesized that polysomes were the sole translation machinery in neurons, with single ribosomes (monosomes) being translationally silent. Recently, however, active translation of mRNA by monosomes in neuronal processes was identified, representing a unique component of local translation in small compartments compared to that in the soma and other cell types^111^.

Additionally, signaling cues and translation factors tightly regulate translation. Several classes of signaling molecules, including neurotransmitters, neurotrophins, and extracellular guidance cues, may induce, suppress, or drive selective translation of certain transcripts^105,112,113^. For example, some mRNA transcripts are transported to distal sites in dormant states and are translated upon arrival by specific stimulation cues^32,114,115^. Others can be partially translated, so that organelle targeting sequences are translated and can participate in the transport of the ribosome nascent chain complex to the targeted organelle^47,116^. Several molecules enable translational repression during transport, including the RBPs cytoplasmic polyadenylation element-binding protein (CPEB) and fragile X mental retardation protein (FMRP)^117^. CPEB is a translation-suppressing protein, which is stimulated upon arrival to activate translation by lengthening mRNA polyA tails^118^. FMRP reversibly regulates translation during transport by repressing translation in its phosphorylated state and enabling it upon dephosphorylation^119^. However, the effect of these translational repression molecules on partially translated transcripts during transport is currently unknown. Another mechanism by which granules may switch between an active transport state and an active translation state is posttranslational modifications and changing of granule components. For example, myelin basic protein transport granules are transported in a translationally repressed state with several RBPs, including hnRNPA2, hnRNPF, and hnRNPE1/PCBP1, but after phosphorylation of hnRNPA2, it and hnRNPE1 leave the granule, allowing phosphorylated hnRNPK to interact with the granule during translation^120–125^. While these myelin-basic protein transport granules may be among the best characterized throughout mRNA transport and translation, the idea of granule remodeling contributing to the transport to translation switch is intriguing and may be particularly disease relevant for co-aggregating granule proteins. Thus, the processes of transport and translation are closely related, although it is still unknown whether transport defects directly lead to translational loss seen in FTD/ALS.

Once transported, translation factors enable protein synthesis of these transcripts, with unique factors for each stage of translation, including eukaryotic initiation factors (eIFs), eukaryotic elongation factors, and eukaryotic release factors^126^. Initiation is the most energy-intensive, rate-limiting step of translation; accordingly, most translational control mechanisms occur at this stage. eIF4E protein, for example, becomes enriched in transport granules under certain conditions, such as neuronal activation, memory formation, and treatment with brain-derived neurotrophic factor (BDNF), increasing translation initiation^127–129^. Conversely, several mechanisms exist to prevent initiation, including the interaction between eIF4E, a cap-binding protein, with eIF4E binding proteins (4E-BPs). 4E-BPs compete with eIF4E for binding at the 5′ mRNA cap site, preventing initiation when bound (reviewed in ref. ^130^). This process can be reversed by certain signaling pathways, which phosphorylate and dissociate 4E-BPs from the binding site, allowing eIF4E to bind and initiate translation. Although these processes are not neurite-specific, some conditions, including neuronal activation and the presence of BDNF, induce the translocation of eIF4E and other translation factors to the axons and dendrites, enabling local translational control^127,128,131,132^. Overall, neuronal translational control is achieved through diverse mechanisms which may either enhance or repress translation under different circumstances.

### Loss of translational control in FTD/ALS: RBPs, repeat expansions, & stress responses

**Fig. 3:**
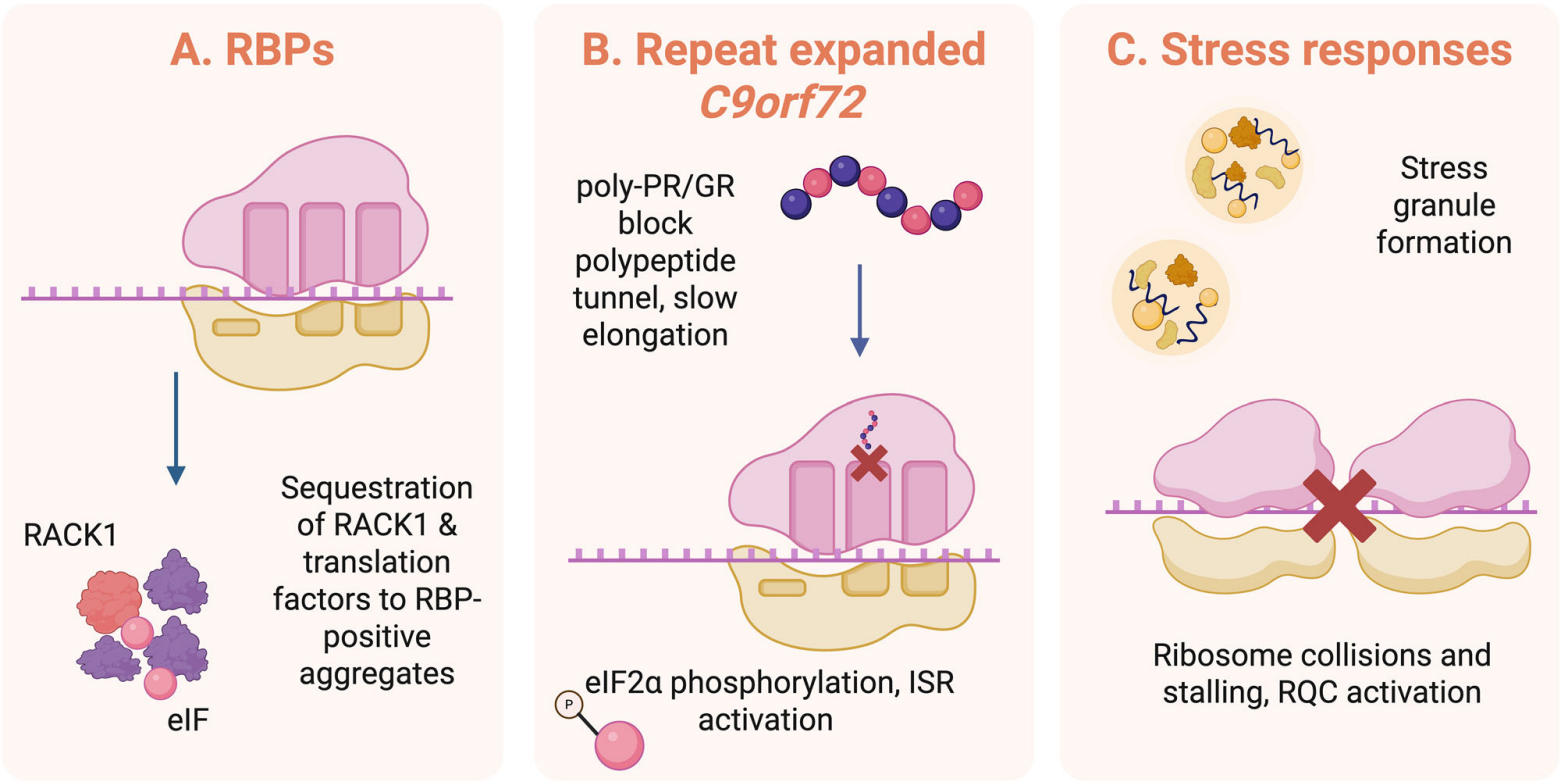
Depiction of the loss of translational control in FTD/ALS. **A** RBP proteinopathy leads to toxic aggregation and sequestration of RBP interactors, including the ribosomal protein RACK1 and translation initiation and elongation factors. **B** *C9orf72* with hexanucleotide expanded repeats forms arginine-rich DPRs like poly-GR/PR, which block the ribosomal polypeptide tunnel and prevent elongation. *C9orf72* also phosphorylates eIF2α, activating the ISR and reducing global translation initiation. **C** Diseased states lead to excessive formation of stress granules, sequestering RBPs, translation factors, and mRNA. Defects in translation elongation can lead to ribosome collisions and stalling, which fail to be resolved by the RQC. Created in BioRender. Craig, E. (2026) https://BioRender.com/1r8ljeg

Several sources contribute to the loss of translational control in FTD/ALS (reviewed in ref. ^133^). First, TDP-43 proteinopathy directly impacts ribosome association and translation of several specific mRNA transcripts in *Drosophila* models^134^. Additionally, TDP-43 overexpression leads to a global reduction in translation^135,136^. In disease this may be due to a close association of TDP-43 with translation machinery, including ribosomal subunits and translation initiation factors (Fig. 3)^137^. Ribosomal transcripts themselves are also reduced in ALS patient-derived cells, which is recapitulated by overexpression of TDP-43^138^. Disruption of these associations likely results in impaired translation of many transcripts, contributing to reduced global translation. This effect may be mediated by the interaction of TDP-43 with the ribosomal protein RACK1, whose overexpression rescues the reduction in global translation caused by TDP-43 overexpression^135^. Further, abnormal phase separation may contribute to TDP-43-mediated translational defects. Normal TDP-43 LLPS is critical for maintaining normal protein synthesis levels, and accumulation and condensate formation of TDP-43 leads to reduced levels of nuclear encoded mitochondrial proteins^139,140^. Similar to TDP-43, expression of mutant FUS leads to reduced protein synthesis globally, as well as deficits in axonal translation in vivo^141^. FUS-mediated disruptions in mitochondrial transport are also linked to a reduction in overall translation^142^. Lastly, both FUS and TDP-43 form co-aggregates with RACK1, which sequester translation machinery and reduce global translation^143^. Although there is currently little work on the direct impacts of hnRNPA1, hnRNPA2, TIA1, and other RBPs on translational defects in FTD/ALS, their roles in translation and association with these diseases suggest a possible role.

Another major contributor to translational dysregulation is the presence of pathological repeat expansions in *C9orf72*. Expanded repeat *C9orf72* transcripts are translated non-canonically by repeat-associated non-AUG (RAN) translation, in which translation initiation occurs without the typical AUG start codon^144,145^. This results in both sense and antisense translation of the *C9orf72* mRNA, producing several dipeptide repeat (DPR) proteins which have well-characterized pathological effects^146,147^. Arginine-rich DPRs, poly-GR and poly-PR, cause a global decrease in protein synthesis, possibly due to reduced ability of translation machinery to access mRNA^148–151^. Poly-GR/PR also interacts with RBPs, ribosomal proteins, and initiation and elongation translation factors, which may contribute to the reduction in translation^148–150,152,153^. Toxic poly-GR/PR-mediated translation repression can be alleviated by expression of a single initiation factor, eIF1A, indicating these DPRs suppress initiation^150^. Further, poly-GR expression disrupts translation elongation and prolongs translation of transcripts with naturally long elongation rates^154^. Additionally, poly-GR/PR were found to block the ribosomal polypeptide tunnel, inhibiting the role of peptidyl-transferase and impairing both initiation and elongation^155^. Finally, DPR-induced activation of the integrated stress response (ISR) may contribute to reduced translation by inhibiting initiation. The ISR is a global translation suppression pathway which decreases protein synthesis under stress conditions by phosphorylating the initiation factor eIF2α^156^. Although the ISR protects cells by maintaining cellular homeostasis during transient periods of stress, overactivation of the ISR is implicated in several neurodegenerative diseases, including FTD/ALS^156^. Poly-GR/PR expression is correlated with cellular and metabolic stress conditions, which lead to ISR activation^157,158^. Additionally, even in the absence of DPR production, antisense repeat expansions in *C9orf72* indirectly lead to ISR activation by promoting eIF2α phosphorylation^159^. Thus, toxic *C9orf72* repeat expansions and translation of DPRs contribute to translational repression in FTD/ALS through multiple mechanisms.

The formation of stress granules and stress responses by the ribosome itself also play a role in impaired translational control in FTD/ALS. Stress granules form in response to external stressors and sequester mRNA, RBPs, translation factors, and other translation machinery. While this protective mechanism allows healthy cells to reduce protein synthesis during stress, prolonged stress granule accumulation and disrupted dynamics are implicated in neurodegeneration^160^. The impact of stress granule formation on neurodegeneration in FTD/ALS is debated, with some studies finding neuroprotective effects of transient stress granule formation, leading to a reduction in toxic TDP-43 aggregation and neurodegeneration^161–163^. Conversely, mutations in TIA1 lead to altered stress granule dynamics, contributing to translation deficits^164^. Similarly, poly-GR/PR disrupt stress granule dynamics, contributing to global translation inhibition^148^, while poly-GR expression reduces translation by promoting stress granule formation and disrupting their disassembly^151^. These conflicting findings may reflect that stress granules have dynamic functions at different time scales. Studies supporting neuroprotective effects of stress granules likely refer to transient models or earlier stages of disease, when stress granule formation may temporarily reduce pathology, whereas research documenting the opposite result show detrimental long-term effects of prolonged stress granule formation and reduced protein synthesis, which may ultimately contribute to disease progression.

Another stress response often excessively activated in FTD/ALS is ribosome stalling. When translation elongation is delayed, either due to RNA damage, stress, ribosome collisions, or other causes, ribosomes may stall and further reduce translation. The ribosome-associated protein quality control system (RQC) detects and dissociates stalled ribosomes (reviewed in ref. ^165^). Increased ribosome stalling and disruption of the RQC itself are both implicated in translational loss in FTD/ALS. Of note, poly-GR/PR blocks the ribosome polypeptide tunnel which prolongs the translation elongation phase, leading to ribosome stalling and RQC activation^155^. This increase in ribosome stalling cannot not be resolved by the RQC^166^. Although no gene directly involved in the RQC has currently been linked to FTD/ALS, mice with mutations in RQC genes exhibit motor and neurological ALS phenotypes, and some RQC mutations are affiliated with other neuromuscular diseases^167,168^. Thus, the RQC may represent an understudied mechanism behind FTD/ALS pathology.

Beyond neurons, other cell types, including oligodendrocytes, microglia, and astrocytes are involved in the disease pathogenesis of FTD/ALS and other neurodegenerative diseases, particularly microglia via their role in neuroinflammation^169,170^. Like neurons, these cells are highly ramified, and there is evidence of long-distance mRNA transport and local translation in glia^171^. Interestingly, mRNA transport and local translation in microglia are critical for phagocytosis^172^ and motility^173^, suggesting that disruption of these processes during disease may increase inflammation. Nonetheless, research connecting the roles of mRNA transport and translation to FTD/ALS outside of neurons is limited, and expanding current knowledge of transport and translation dynamics to new cell types may provide a more comprehensive understanding of these diseases.

## Mechanistic interplay and therapeutic targeting

While RNA transport and translation represent distinct but interdependent processes, multiple cellular pathways are linked to both, including nucleocytoplasmic transport (NCT), pathological phase transitions, and posttranslational modifications. Here, we summarize these processes in disease contexts and highlight therapeutic targeting strategies correcting them.

### Mechanistic interplay: nucleocytoplasmic transport, phase transitions, posttranslational modifications, and other mechanisms

NCT is the process of trafficking molecules through nuclear pores via the nuclear pore complex (NPC). The process is tightly regulated by nucleoporins, importin and exportin nuclear transport receptors, and biochemical pathways including the Ran-GTP cycle, all of which enable the accurate trafficking of many nuclear cargoes (reviewed in ref. ^174^). NCT is vulnerable to diseased states and disruption to this process has deleterious effects on transport, localization, and translation^175^. In FTD/ALS, NCT dysregulation is linked to pathology in multiple RBPs and *C9orf72* repeat expansions. Hexanucleotide expanded repeats in sense strands of mutated *C9orf72* RNA and poly-PR interact directly with Ran-GTP cycle proteins, nuclear envelope proteins, and other components of NCT machinery^176–178^. Furthermore, loss of the healthy C9orf72 protein or expression of hexanucleotide repeat expansions leads to disruptions in NCT by perturbing the Ran-GTPase gradient, increasing the sequestration of importins and nucleoporins to pathological aggregates, and altering interactions with NPC proteins^179,180^. These *C9orf72*-associated perturbations lead to faulty RNA transport and translation by altering the import and export of transcripts and translation factors through the NPC. In addition, several FTD/ALS-associated RBPs are involved in NCT disruption^179^. Mutated FUS, for example, interacts aberrantly with nucleoporins, leading to an overall reduction in NCT^181^. FUS mutations, particularly in the C-terminal nuclear localization signal, do not show reductions in phase separation upon interaction with transportin (TNPO1) as happens to wildtype FUS, and TNPO1 is aggregated in FTD patient tissue, suggesting that these interactions are crucial for maintaining the function of both proteins^182^. An in vitro study also revealed negative impacts of mutated TDP-43 on NCT, sequestering NPC components and NCT machinery into TDP-43 positive aggregates, causing mislocalization of transport factors and nucleoporins, and interfering with RNA export^183^. NCT dysfunction also causes mislocalization of FTD/ALS-associated RBPs themselves, leading to the formation of RBP-positive toxic aggregates in the cytoplasm^183^. Further, nuclear pore injury is observed in iPSC-derived neurons from sporadic and familial ALS cases, demonstrating the importance and prevalence of proper NCT, and reducing the nuclear pore quality control protein, CHMP7, rescues some of these defects^184,185^. Given the association between RBPs and translational machinery, these aggregates may also sequester key proteins involved in translational control, representing another mechanism by which NCT disruption contributes to translation defects in FTD/ALS.

FTD/ALS are characterized by altered phase transitions (reviewed in ref. ^186^). This leads to the sequestration of translation factors and RBPs, including TDP-43, FUS, hnRNPA1, and hnRNPA2, into solid aggregates, disrupting the transport and translation-associated functions of these proteins^76,89,187,188^. Aberrant interactions between these RBPs, including ANXA11/TDP-43, TAF15/FUS, and hnRNPA1/TDP-43, lead to the formation of co-aggregates in FTD/ALS, which may increase their pathological interactions and promote disease states^68,189,190^. Dissolving these aggregates may both improve granule dynamics and reduce the frequency of deleterious interactions, while also alleviating added stress on the proteostasis systems. Posttranslational modifications (PTMs) regulate these phase transitions and may be promising therapeutic targets for neurodegenerative disease. TDP-43 becomes hyperphosphorylated in FTD/ALS and phosphorylation modulates TDP-43 LLPS, although its role in this process is debated (reviewed in ref. ^191^). One study has indicated that TDP-43 phosphorylation rescues TDP-43 pathology^192^ and improves liquid-like dynamics of TDP-43, leading to the hypothesis that hyperphosphorylation is protective. In contrast, other studies suggest that TDP-43 phosphorylation impairs LLPS and promotes aggregation in vitro^193–195^. This contradictory result may reflect that TDP-43 phosphorylation has distinct impacts on phase separation under different conditions, suggesting that TDP-43 phosphorylation might be too complex as a therapeutic target and work should focus on phosphorylation of other proteins or other PTMs of TDP-43, like polyADP-ribosylation^196^. hnRNPA2 also contributes to transport granule dynamics, regulating the repression of translation during transport and stimulating translation when phosphorylated at its destination^121,125^. This hnRNPA2 phosphorylation reduces phase separation and prevents aggregation in diseased states, mitigating neurodegeneration^197,198^ while also enabling exchange of granule components to help initiate translation^121–123,125,197,198^. FUS phosphorylation similarly acts to regulate phase separation, prevent protein aggregation, and mitigate neurodegeneration^199,200^. Furthermore, methylation of FUS modulates phase transitions, and FUS hypomethylation, observed in FUS-FTD, increases phase transitions and aggregation^93^. Finally, persistent or toxic stress granules associated with disease are ubiquitinated by multiple pathways to promote their clearance^201,202^. Ubiquitination and other PTMs, like methylation, PARylation, acetylation, and glycosylation, have not been as well studied as phosphorylation, but influence phase transitions, while RNA modifications like m6A add an additional level of complexity to this equation but have not yet been studied (reviewed in ref. ^41^).

Several additional mechanisms, including RNA processing, mitochondrial dysfunction, and proteostasis, may also contribute to disease pathology. Recent transcriptomic studies on FTD/ALS patients have revealed disruptions to transcripts involved in RNA metabolism, splicing, and polyadenylation, and may be an additional promising direction for future work^203,204^. Changes in splicing may make non-functional or misfolded proteins, leading to additional stress, or cause the transcript to be degraded via nonsense-mediated decay. If these splicing changes affect zipcodes or targeting sequences, then these transcripts may not be properly localized, causing additional downstream issues. Pathological TDP-43 also mediates alternative polyadenylation in FTD patients and hyperactivates processing bodies, regulators which mediate mRNA degradation and decay^205,206^. Multiple aspects of mitochondrial function are disrupted in FTD/ALS, including its transport and energy production. As motor proteins rely on ATP produced by mitochondria, trafficking of mRNA along the axon may be reduced when mitochondrial function is impaired in disease^207^. The co-trafficking of mRNA and mitochondria may also be disrupted in disease, either due to changes in mitochondrial function or mRNA transport. Similar effects may be observed for translation, as translation is one of the most energetically costly functions of the cell. Finally, disruption to proteostasis, beyond aggregation, particularly the ubiquitin proteasome system, may contribute to FTD/ALS pathology by contributing to protein misfolding and abnormal ubiquitination^208^. The unfolded protein response may not function normally in FTD/ALS, but activation of it may improve disease course^209,210^. Targeting misfolded proteins has been tested in many pre-clinical models and seems to improve symptoms^211^. These defects may be additional potential targets to harness for future therapies.

### Therapeutic targeting: current progress and future directions

Despite advances in understanding disruptions in RNA transport and translation in FTD/ALS, there has been limited success in developing strategies correcting these defects. A leading technique is gene knockdown via antisense oligonucleotides (ASOs), which are short, single-stranded regions of DNA which can bind, regulate, and degrade specific RNA sequences of interest^212^. A leading target is repeat-expanded *C9orf72*, as it is the most common genetic cause of FTD/ALS. ASO knockdown of repeat-expanded *C9orf72* in iPSCs and mouse models reduces multiple components of RNA toxicity^213–215^, and *C9orf72*-mediated defects in NCT and TDP-43 localization have been corrected by ASOs in *Drosophila* models^176^. ASOs also reduce the expression of toxic poly-PR/GR and sense *C9orf72* RNA in mice, although the specific effects of knockdown on transport and translation are less well defined in these models^216^. Despite these promising advances, ASOs have several notable limitations, and reducing toxic pathology while retaining normal *C9orf72* function is difficult. Indeed, testing of these ASOs in patients is still early, and two recent clinical trials have shown limited success^217^. The effect of these ASOs on the disruptions in mRNA transport and local translation associated with *C9orf72* repeat expansions has not yet been evaluated. Similarly, ASOs targeting Ataxin-2 increase lifespan and reduce TDP-43 pathology in mice, although clinical trials of the same ASOs were again unsuccessful^218^. Beyond ASOs, several other strategies are in the early stages of preclinical testing to treat FTD and ALS. A recent experiment in *C9orf72*-ALS patient-derived neurons and *Drosophila* indicated a neuroprotective effect of a gene therapy technique inhibiting the nuclear export of *C9orf72* RNA^219^. Similarly, epigenetic small molecules targeting toxic DPRs yielded a reduction in DPR cytotoxicity and mitigated NCT dysfunction in vitro^220^. However, these techniques have yet to be studied in animal and human models.

Altogether, basic advances in FTD/ALS research and early preclinical efforts hold promise, but more work is needed to translate these findings into meaningful treatments or diagnostics. A promising future therapeutic target may be the prevention of toxic interactions between RBPs. Pathological interactions between RBP pairs, particularly those found in aggregates in FTD/ALS patients like ANXA11/TDP-43 and TAF15/FUS, contribute to disease phenotypes^68,190^. A method of dissolving these aggregates may both improve granule dynamics and reduce the frequency of deleterious interactions. Similarly, taking advantage of key regulators of translational control and phase separation, like RBP phosphorylation, could improve patient outcomes. Given that altering the phosphorylation of TDP-43, FUS, and hnRNPA2 often has protective effects on toxic phase transitions and neurodegeneration, targeting the kinases and phosphatases that act on involved in the phosphorylation of these RBPS could reduce toxicity in patients. A notable limitation of this approach, however, is that TDP-43 is a critical RBP interacting with many transcripts, and altering its phosphorylation could cause detrimental off-target effects. Additionally, given that several FTD/ALS-associated mRNAs are involved in RNA processing, studying these transcripts and their contribution to disease further may inform their therapeutic potential^205,206^. Targeting LLPS using regulatory compounds, small RNA oligonucleotides, or modulating RNA G-quadruplexes has also been proposed^221–223^ and shows promise in pre-clinical studies. Indeed, a recent pre-clinical study showed that short RNAs dissolve TDP-43 condensates and aggregates, restore nuclear localization in iPSC-derived neurons, and restore TDP-43 function in a mouse model^224^. Several more studies have tested modulation of the ISR as a therapeutic strategy in FTD/ALS, but this may need to be a personalized therapy, as each model or genetic mutation shows different effects on the ISR (reviewed in ref. ^225^). Furthermore, the use of microtubule-stabilizing agents has been proposed to improve axonal transport and reduce neurodegeneration in multiple diseases^226^, while translation deficits have been corrected by overexpression of RACK1 and partial inhibition of the ISR using the kinase PERK in preclinical models^135,227^. Finally, NIRs can both prevent and reverse RBP fibrilization, making them an attractive candidate for drug development. Indeed, an adeno-associated virus delivery method using Kapβ2 has been proposed as a potential therapy, although this method has yet to be tested in the clinic^228^.

Further, the potential for mRNA transport and local translation defects as biomarkers of disease has not yet been extensively explored. Elevated neurofilament light (NfL) in blood and cerebrospinal fluid is currently being used, but primarily tracks neuron damage and cannot currently distinguish between different types of neurodegeneration. Alternatively spliced RNAs and the resulting proteins have been proposed as potential biomarkers of TDP-43 dysfunction, but more work is needed to determine the prevalence of these splicing changes in patients across disease stages. mRNA transport might be an attractive potential biomarker, especially as neuronal extracellular vesicles can originate from both dendrites and axons as well as the cell body^224^, however, more work is needed to identify any changes in extracellular vesicle RNA due to transport defects. Local protein translation may be a more difficult biomarker to parse, but it may be possible to identify the effects of disrupted translation as a biomarker, either altered ribosome levels or changed expression of certain proteins that could be identified in cerebrospinal fluid or extracellular vesicles.

Several recent and ongoing clinical trials are testing drugs treating FTD/ALS. A recent trial of a tau ASO for Alzheimer’s disease yielded a reduction in tau pathology, suggesting that ASOs can be effective in treating neurodegenerative disease, but perhaps the correct targets to treat FTD/ALS have not yet been identified^229^. Two additional ASO trials are ongoing, targeting SOD1^230^ and FUS^231^, which may hold promise for genetic forms of ALS. Furthermore, a personalized ASO targeting TDP-43 in a single patient has recently been completed, although the results of this study have not yet been published^232^. siRNAs, therapies similar to ASOs which knock down specific target RNAs, are also currently being tested to treat patients with mutations in SOD1^233^ and FUS^234^. Another major class of therapies currently in clinical development is partial inhibition of the ISR using activators of eIF2B^235,236^ and small molecules^237^. Finally, a current trial is investigating the efficacy of a drug to regulate RAN translation in *C9orf72* FTD/ALS^238^. Targeting disrupted facets of translation and transport has the potential to improve transport and translation outcomes and alleviate disease pathologies or diagnose disease states, although clinical studies are still in early stages.

## Conclusions and future directions

Substantial work has begun to elucidate the mechanisms contributing to pathology in FTD/ALS, including disruptions to RNA localization, neuronal transport, and local translation. We expect the field will continue to expand upon recent developments in identifying zipcode regions, localization motifs, and RBP interactomes to better characterize the local transcriptome and its disruption in disease states. Furthermore, while the roles of RBPs, *C9orf72*, and the RQC in translational control in FTD/ALS are strongly supported, their direct interactions with translation machinery could be further explored. Lastly, we expect future research to closely study the mechanisms yielding toxic phase transitions, LLPS, and modulation by PTMs, and testing proposed methods targeting them. Correcting these and other disruptions by targeting mutations or proteinopathies in relevant RBPs, *C9orf72* mutations and toxic DPRs, and NIRs, posttranslational modifications, and other regulators of phase transitions may offer promising targets for desperately needed therapies. The strong evidence supporting roles for neuronal transport and translation in FTD/ALS makes the topic a compelling candidate for future research. Diving deeper into the mechanisms described here and building upon current translational work may form a crucial foundation informing future therapeutic development.

## Data Availability

No datasets were generated or analysed during the current study.
